# Iatrogenically Acquired Mycobacterium abscessus Infection in an Indwelling Intercostal Drainage In Situ in a Patient With Alcoholic Liver Disease and Bilateral Hepatic Hydrothorax: A Report of a Rare Case

**DOI:** 10.7759/cureus.59626

**Published:** 2024-05-04

**Authors:** Rahul Ranjan, Jayanthi Gunasekaran, Raunak Bir, Umesh Kumar, Rajiv M Gupta

**Affiliations:** 1 Department of Microbiology, Employees' State Insurance Corporation (ESIC) Medical College and Hospital, Faridabad, IND

**Keywords:** combination therapy, iatrogenic, ntm, macrolide, hepatic hydrothorax, pigtail catheter, pleural tap, pleural fluid, subspp abscessus, mycobacterium abscessus

## Abstract

A 47-year-old male, a known case of alcoholic chronic liver disease with portal hypertension, presented with complaints of abdominal distension and shortness of breath. A provisional diagnosis of ethanol-related compensated chronic liver disease (CLD) with portal hypertension and splenomegaly, gross ascites with bilateral hepatic hydrothorax was made. The left-sided pleural effusion subsided after three pleural taps, but the right-sided effusion kept refilling even after four to five days of repeated therapeutic taps, so a pigtail catheter was left in situ. The pleural fluid was sent for culture which did not grow any pathogenic organisms. Cartridge-based nucleic acid amplification tests where *Mycobacterium tuberculosis* complex (MTBC) was not detected, Ziehl-Neelsen staining was done in which acid-fast bacilli were not seen, and cytology was done where no malignant cells were seen. The patient was discharged with the pigtail in situ on the right side and, after 20 days, the patient again presented with shortness of breath, and imaging revealed moderate right-side pleural effusion. Draining of pleural fluid was done and sent for investigation which again revealed no infective etiology. The patient was admitted to the hospital for one month as the right-sided effusion did not resolve. Suddenly, the patient developed shortness of breath, and a chest X-ray was done, which showed pigtail blockage; pigtail flushing was done, and the bag was drained. The patient was empirically started on IV meropenem 500 mg TID, IV teicoplanin 400 mg BD, and inj polymyxin B 500,000 IU IV BD. The pleural fluid was sent continuously for investigation for the first two months which again did not reveal any infective etiology. After two months of pigtail in situ, the pleural fluid was sent for CBNAAT where MTBC was not detected, and ZN stain showed smooth acid-fast bacilli. The sample was cultured, and it grew acid-fast bacilli in 72 hours on blood agar, MacConkey agar, and Lowenstein-Jensen media. A line probe assay done from the isolate revealed it to be *Mycobacterium abscessus* subsp. abscessus which was resistant to macrolides and sensitive to aminoglycosides. *Mycobacterium abscessus* subsp. abscessus was isolated from repeated cultures of pleural fluid, and the patient was advised on a combination treatment of amikacin, tigecycline, and imipenem. The patient was discharged with the indwelling pigtail with the advised treatment; unfortunately, we lost patient follow-up as the patient never returned to us.

## Introduction

Rapidly growing mycobacteria (RGM) are widespread non-tuberculous mycobacteria (NTM) species found in soil, dust, rocks, and water. They exhibit visible growth on solid media within a seven-day period [1]. RGM, particularly *Mycobacterium abscessus*, *Mycobacterium fortuitum*, *Mycobacterium chelonae*, and *Mycobacterium mucogenicum*, are increasingly recognized as causative agents across a broad range of diseases. These include pulmonary, skin, soft tissue, and disseminated infections [1].

After *M. fortuitum*, the *M. abscessus* complex (MABC) is the second most common RGM species in clinical specimens [2]. MABC stands out as the most pathogenic among the RGM. Specifically, MABC exhibits both intrinsic and acquired resistance to a wide range of anti-mycobacterial agents, including macrolides [3]. MABC can lead to infections affecting nearly all organs, although it predominantly involves the lungs, skin, and soft tissue [4]. MABC is the predominant causative agent of pulmonary disease among RGM, accounting for 3% to 13% of all nontuberculous mycobacterial pulmonary diseases (NTM-PD) [5]. In regions with a high burden of tuberculosis (TB), NTM-PD is frequently misdiagnosed as multidrug-resistant tuberculosis (MDR-TB) [6].

Infections resulting from MABC pose greater treatment challenges due to their antimicrobial drug resistance [7]. MABC consists of three subspecies: *M. abscessus* subsp. abscessus, *M. abscessus* subsp. massiliense, and *M. abscessus *subsp. bolletii. Recent studies have highlighted concerning treatment outcomes associated with macrolide resistance. This resistance can occur due to either mutational or inducible mechanisms, often linked to the presence of a functional erm (41) gene in *M. abscessus *subsp. abscessus and bolletii [8]. According to IDSA 2020 guidelines, it is recommended to employ a treatment regimen with at least three active drugs for macrolide-susceptible *M. abscessus *diseases, and whenever feasible, use a minimum of four drugs for macrolide-resistant diseases [8].

## Case presentation

**Figure 1:**
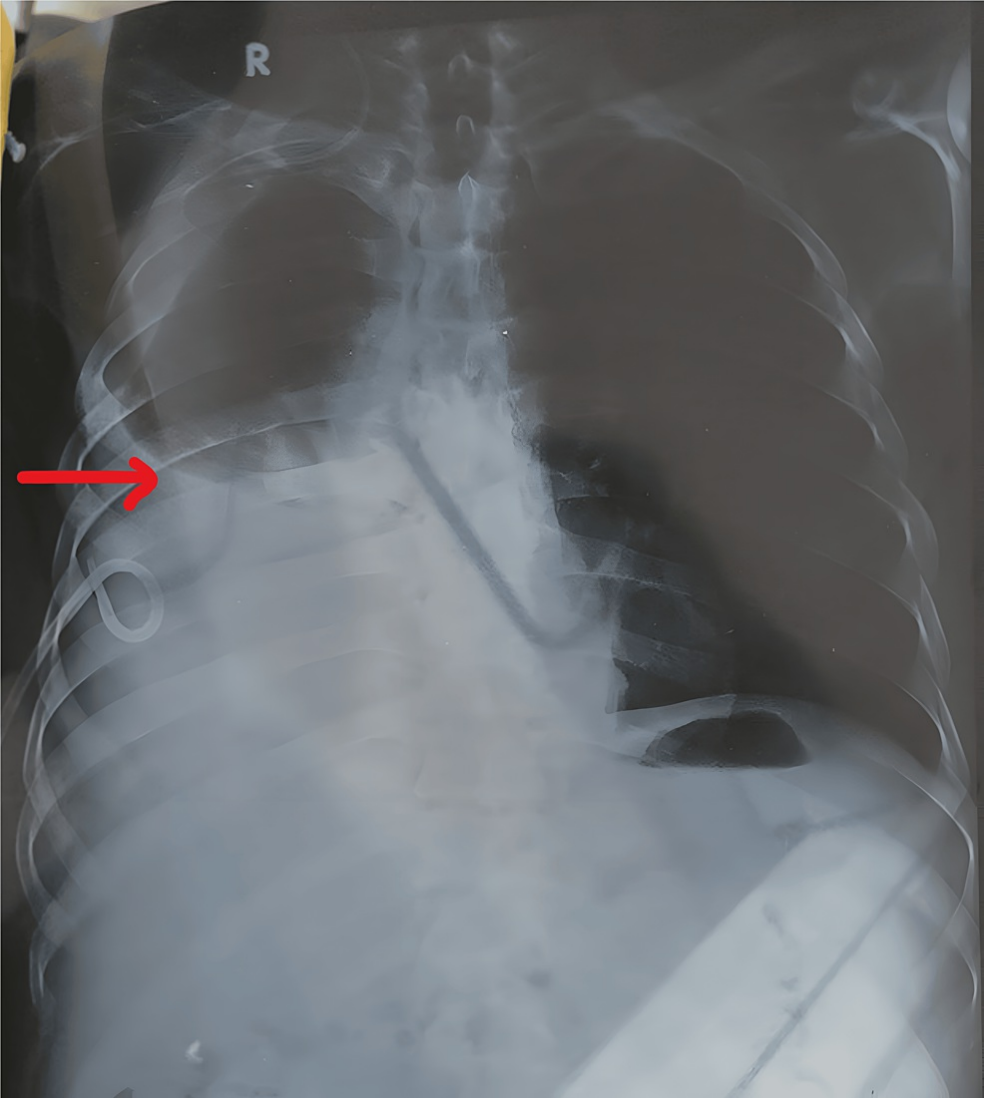
X-ray showing moderate pleural effusion on the right side. X-ray showing moderate right-side pleural effusion with pigtail catheter, and mild left-side pleural effusion showing blunting of costo-phrenic angle.

A 47-year-old male, a known case of chronic liver disease (CLD) with portal hypertension, came to the Medicine Outpatient Department with complaints of abdominal distension and shortness of breath, fever associated with headache, and dyspnea on exertion for five days. There was no history of similar complaints in the past. There was no history of hypertension, diabetes mellitus, tuberculosis, or epilepsy. On examination, the heart rate was 82/minute, blood pressure was 93/60 mmHg, and respiratory rate was 32-35/minute. On auscultation of the chest, bilateral decreased air entry was found, more so in the right lung, and dullness to percussion on the right side. A chest X-ray was performed, suggestive of moderate pleural effusion on the right side (Figure 1).

The patient was admitted, and a left-sided pleural tap was performed, draining approximately one liter of pleural fluid. A pigtail catheter was placed for the repeated filling of right-sided pleural effusion even after four to five days of therapeutic tap.

A provisional diagnosis was made of ethanol-related compensated CLD with portal hypertension and ascites, splenomegaly, gross ascites with hepatic hydrothorax. Other investigations revealed HBsAg, anti-HCV, and HIV were negative, blood hemoglobin was 8 g/dL, and total leukocyte count was 3130/µL.

The patient was discharged with the pigtail in situ on the right side. After 20 days of discharge from the hospital, the patient again came to the hospital with breathing difficulty and was admitted to the ICU with the pigtail in situ; around 500 mL of pleural fluid was drained from the right side. The pleural fluid was transudative in nature, and the patient was empirically started on inj meropenem 500 mg IV TID, inj teicoplanin 400 mg IV BD, and inj polymyxin B 750,000 IU IV BD.

On investigation, blood hemoglobin was 8.5 g/dL, blood urea was 78 mg/dL, serum creatinine was 1.5 mg/dL. Total bilirubin was 1.66 mg/dL, direct bilirubin was 1.03 mg/dL, and indirect bilirubin was 0.63 mg/dL. Serum sodium/potassium levels were 134/4 mEq/L.

After two days of admission, on examination, bilateral crepitations were heard in both lungs; one liter of fluid was drained. CBC, electrolytes, and liver function tests were done throughout the admission period, and the details are compiled in Table 1.

**Table 1:** Sequence of investigations/events and their details After one week, the patient was discharged, a line probe assay was done, and the isolate was identified as *Mycobacterium abscessus *complex. S. urea, serum urea; S. creatinine, serum creatinine; CBNAAT, cartridge-based nucleic acid amplification test; ZN, Ziehl-Neelsen; MTBC, *Mycobacterium tuberculosis* complex; AFB, acid-fast bacilli; ICD, intercostal drain.

DOA	Hb, mg/dL	S. urea, mg/dL	S. creatinine, mg/dL	Albumin, g/dL	Globulin, g/dL	Total bilirubin, mg/dL	Direct bilirubin, mg/dL	Indirect bilirubin, mg/dL	Sodium, meq/L	Potassium, meq/L	Total leucocytes/µL	Blood culture	Pleural fluid culture	CBNAAT and ZN staining
First admission	8	78	1.8	2.4	2.7	1.66	1.06	0.60	134	4	3130	Sterile	Sterile	MTBC not detected and AFB not seen
Second admission Day 1	8.5	75	1.7	2.7	2.4	2.7	1.5	1.2	132	4.2	3770	Sterile	Sterile	MTBC not detected and AFB not seen
Day 3	8.3	69	1.6	2.8	2.3	2.72	1.50	1.2	130	4.4	3440	Not Done	Sterile	MTBC not detected and AFB not seen
Day 4	8.2	64	1.6	2.8	2.3	2.1	1.2	0.9	131	4.2	3470	Sterile	Sterile	MTBC not detected and AFB not seen
Day 8	8	61	1.5	2.7	2.4	2.2	1.2	1.0	130	4.1	3300	Sterile	Sterile	MTBC not detected and AFB not seen
Day 10	8	63	1.5	2.7	2.4	2.3	1.2	1.1	132	4.2	3400	Not done	Sterile	MTBC not detected and AFB not seen
Day 12	7.9	64	1.5	2.8	2.3	2.2	1.2	1.0	134	4.4	3450	Sterile	Sterile	MTBC not detected and AFB not seen
Day 17	7.8	62	1.4	2.8	2.3	2.1	1.2	0.9	135	4.3	3470	Sterile	Sterile	MTBC not detected and AFB not seen
Day 25	7.8	61	1.4	2.8	2.3	2.4	1.2	1.2	132	4.2	3480	Sterile	Sterile	MTBC not detected and AFB not seen
Day 32	7.8	64	1.4	2.8	2.3	2.4	1.2	1.2	131	4.1	3500	Sterile	Sterile pigtail was blocked and flushing was done	MTBC not detected and AFB not seen
Day 40	7.8	62	1.4	2.8	2.3	2.3	1.2	1.1	132	4.4	3550	Sterile	Sterile	MTBC not detected and AFB not seen
Day 45	7.8	62	1.4	2.9	2.2	2.4	1.2	1.2	134	4.3	3560	Sterile	ICD was inserted	MTBC not detected and AFB not seen
Day 52	7.8	64	1.3	3.0	2.1	2.3	1.2	1.1	132	4.2	3600	Sterile	Candida ciferrii isolated	MTBC not detected and AFB seen (NTM was diagnosed)
Day 65	7.8	64	1.3	3.0	2.0	2.3	1.2	1.1	130	4.1	3650	Sterile	Not done	MTBC not detected and AFB not seen

On day 13 of ICU admission, grade IV esophageal varices were seen on upper GI endoscopy, and esophageal variceal ligation (EVL) was performed. Meanwhile, the culture of the pleural fluid did not grow any bacterial pathogens. No growth was obtained from aerobic culture of the blood. Meanwhile, one liter of fluid was drained and pigtail dressing was done.

On day 25 of ICU admission, the patient complained of shortness of breath for one day. On auscultation, bilateral air entry was present, but decreased air entry was noted on the right side. Bilateral wheeze was present. A chest X-ray showed pleural tube blockage. Pigtail flushing was done, the bag was changed, and 200 mL of pleural fluid was drained. The patient was also transfused with two units of fresh frozen plasma.

**Figure 2:**
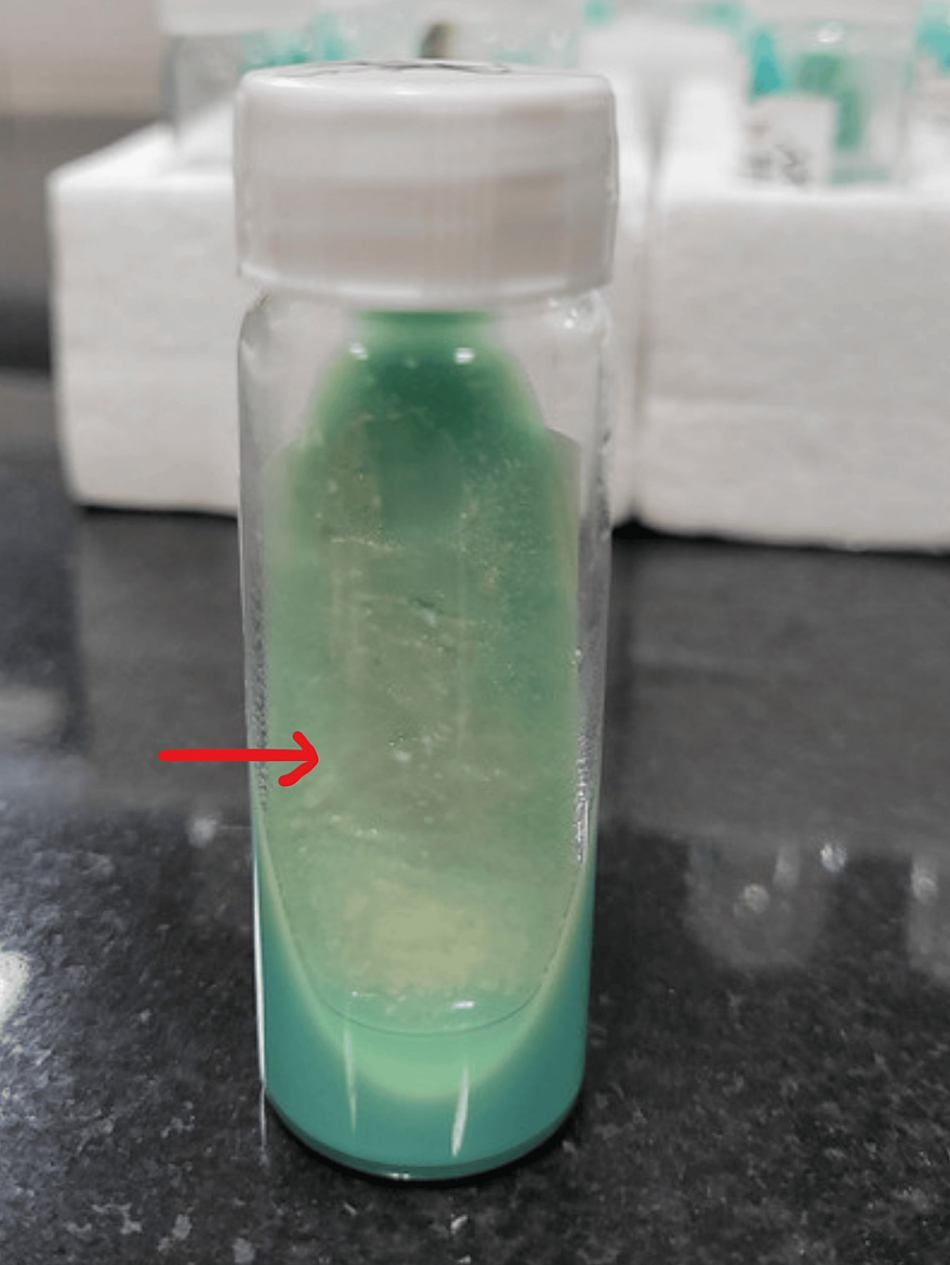
Lowenstein-Jensen medium showing smooth moist colonies suggestive of non-tuberculous mycobacteria.

**Figure 3:**
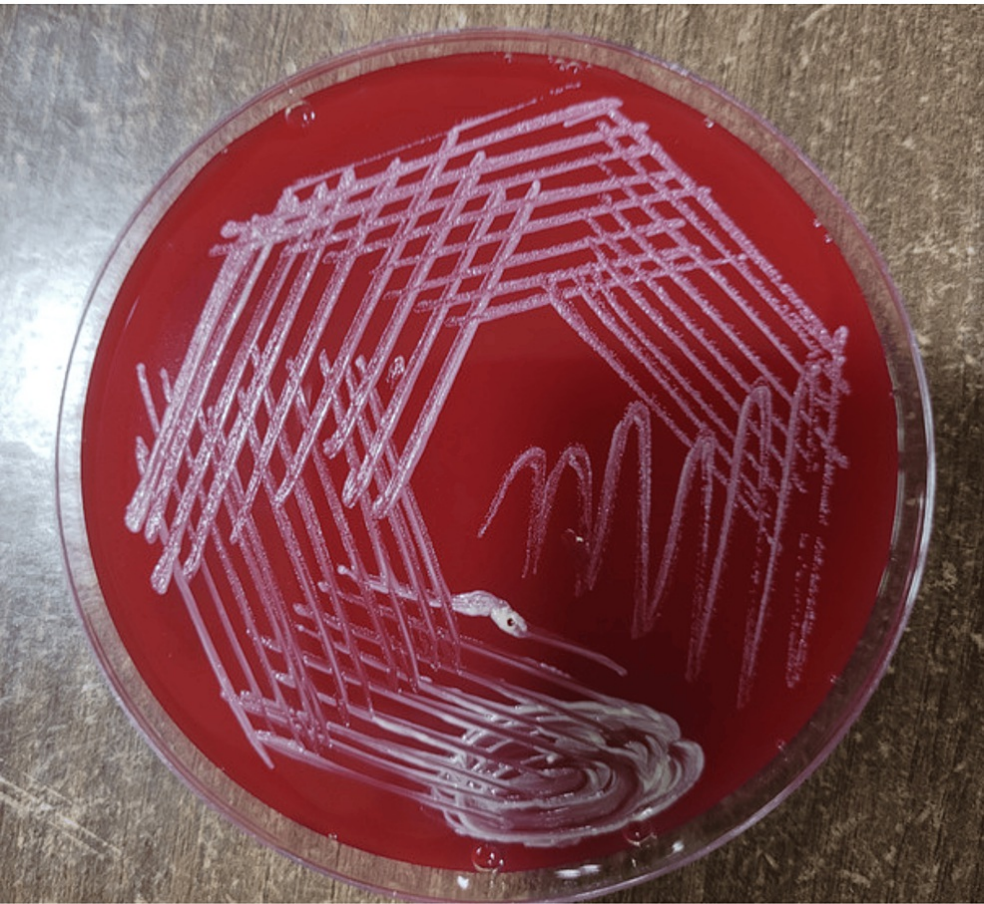
Blood agar showing the smooth colonies of non-tuberculous mycobacteria.

**Figure 4:**
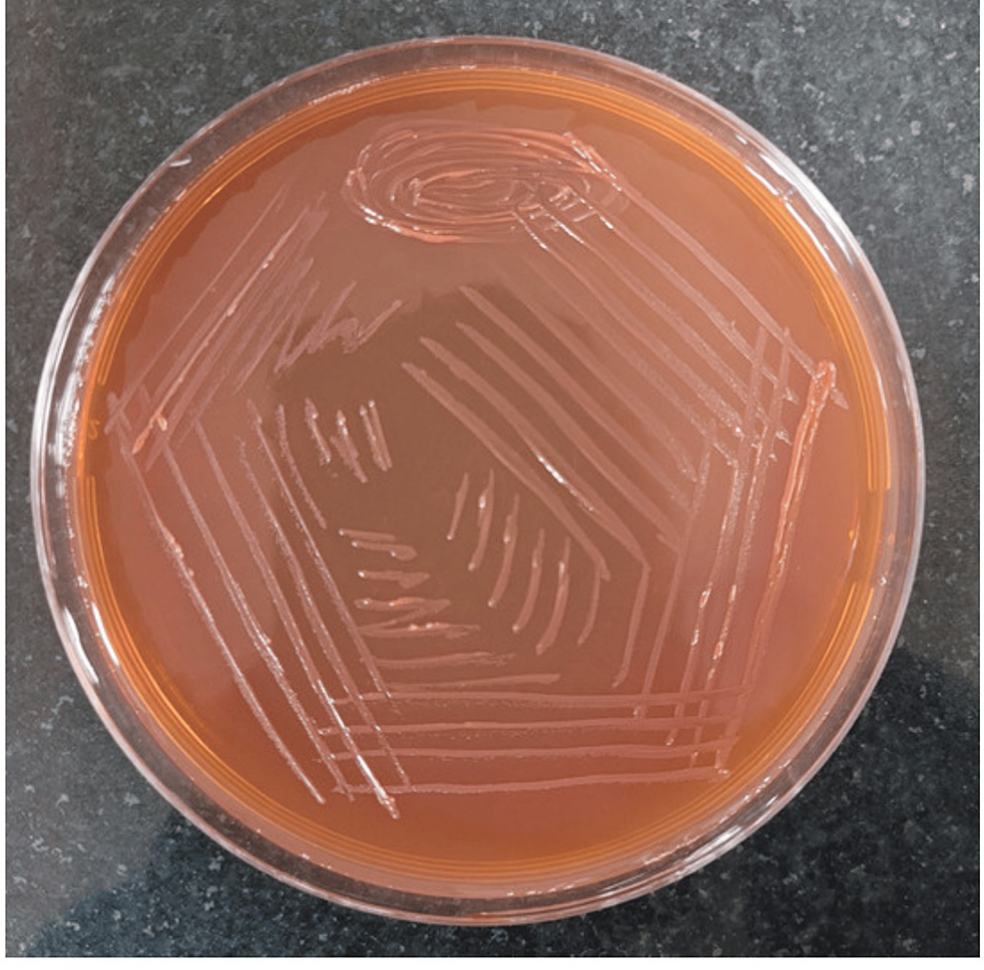
MacConkey agar showing the smooth colonies of non-tuberculous mycobacteria.

On day 45 of ICU admission, the patient was hemodynamically stable and was shifted to the ward. After the patient was shifted to the ward with the pigtail in situ on the right side, it was found that bilateral air entry was present but decreased on the right side of the lung. An X-ray showed a massive pleural effusion on the right side. CBNAAT from pleural fluid was negative for *Mycobacterium tuberculosis* complex. However, the Ziehl-Neelsen stain demonstrated smooth acid-fast bacilli. A diagnosis of NTM was made. The sample grew NTM in three to four days on Lowenstein-Jensen media (Figure 2), blood agar (Figure 3), and MacConkey agar (Figure 4). Consecutive two pleural fluid samples grew NTM, confirming the presence of NTM in the sample.

A provisional diagnosis was made of decompensated CLD with portal hypertension, ascites, and hepatic hydrothorax (right-side massive pleural effusion). The pigtail was present in situ for more than two months.

On day 56 of admission, a 24G intercostal drain (ICD) was inserted at the site of the pigtail insertion on the right side under aseptic conditions to drain the pleural effusion. Pleural fluid examination revealed no malignant cells and was transudative in nature. A chest X-ray revealed a right-sided pleural effusion. Massive pleural fluid of 1,000 mL and 750 mL was drained on subsequent days.

Subsequently, the patient complained of chest pain and pain in the abdomen. On examination, a leak was observed from the ICD site and 450 mL of pleural fluid was drained.

**Figure 5:**
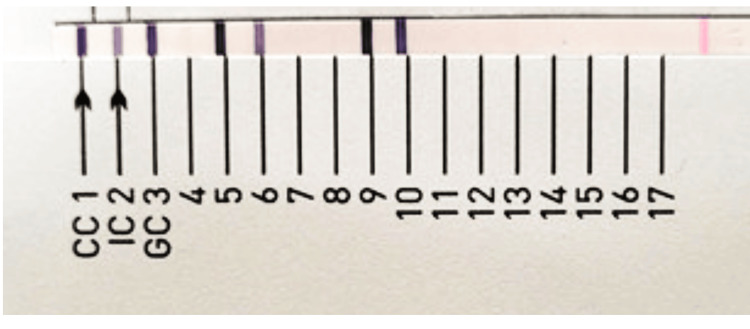
Line probe assay showing band pattern matching with Mycobacterium abscessus complex. Line probe assay using GenoType NTM CM VER 2.0 showing band pattern matching with *Mycobacterium abscessus *complex.

The patient was discharged after 65 days of admission with the final diagnosis of CLD with portal hypertension, ascites, right hepatohydrothorax with ICD in situ on the right side, esophageal varices with EVL done, and iatrogenically acquired non-tuberculous mycobacterial infection. He was discharged with the following advice: Tab rifaximin 550 mg BD, Tab lasilactone 20/50 mg OD, syrup lactulose 30 mL TDS, Tab ondansetron 4 mg SOS, Tab azithromycin 500 mg OD for two days, Tab ursodeoxycholic acid 150 mg BD, Tab carvedilol 3.125 mg OD, Tab pantoprazole 40 mg OD, and Tab paracetamol 500 mg SOS. After one week of the patient being discharged, *Mycobacterium abscessus* complex was detected from pleural fluid by line probe assay (LPA) test using GenoType CM direct VER 2.0 (Figure 5).

**Figure 6:**
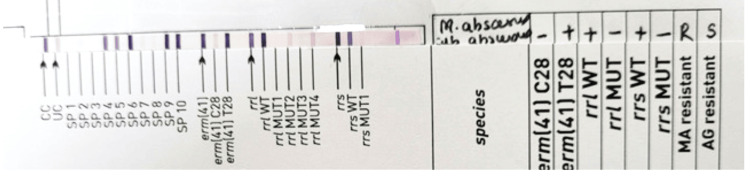
Line probe assay showing the band patterns matching with Mycobacterium abscessus subspp. abscessus, demonstrating a resistant pattern to macrolides and a sensitive pattern to aminoglycosides. The line probe assay testing by GenoType NTM DR ver 1.0 shows band patterns matching *Mycobacterium abscessus *subsp. abscessus, displaying a resistant pattern to macrolides (presence of the erm(41) gene at T28 and absence of mutant bands, with the presence of all wild-type bands of the rrl gene) and a sensitive pattern to aminoglycosides (absence of mutant bands and presence of all wild-type bands of the rrs gene).

The isolate was subjected to LPA testing using GenoType NTM DR VER 1.0. It was found that *Mycobacterium abscessus *subsp. abscessus was resistant to macrolides and sensitive to aminoglycosides, as shown in Figure 6. The patient was advised to undergo combination treatment consisting of amikacin, tigecycline, and imipenem.

As we lost follow-up with the patient, further treatment for NTM was not administered according to the susceptibility results.

## Discussion

The increasing utilization of IPCs has ushered in a new era in managing recurrent symptomatic pleural effusions. These soft silicone tubes allow individuals to better manage shortness of breath from recurrent malignant pleural effusions, even in the comfort of their homes [9].

In various reported (typically small) series, the incidences of IPC-related pleural infections have been observed to range from 0% to 12% [9]. Research has demonstrated that pleural infections with positive cultures are linked to increased mortality rates, longer hospital stays, and poorer surgical outcomes [10]. In a comprehensive multicentric review, which included 1,021 patients with IPCs from 11 centers across Europe, North America, and Australia, researchers found that the infection rate associated with IPCs was remarkably low, standing at only 4.9% of patients, and the overall mortality risk was approximately 0.3%. *Staphylococcus aureus* was the causative organism in 48% of cases, followed by *Pseudomonas aeruginosa* and Enterobacteriaceae [11].

Our case report highlighted an incidence of an iatrogenically acquired *Mycobacterium abscessus* infection in an indwelling intercostal drainage in situ in a chronic alcoholic liver disease patient. To the best of our knowledge, this is the first case report of an iatrogenically acquired *Mycobacterium abscessus* infection in an indwelling intercostal drainage. The patient had an indwelling catheter for about two months. There was a history of catheter blockage and subsequent flushing, which might have led to the introduction of *Mycobacterium abscessus* into the pleural cavity.

*Mycobacterium abscessus* and its subspecies, including *M. abscessus*, *M. bolletii*, and *M. massiliense*, stand out as the predominant culprits responsible for pulmonary diseases caused by RGM [12]. Our case represents the first documented report of isolating *Mycobacterium abscessus* from the pleural fluid of a patient with an indwelling intercostal drainage catheter.

Antibiotic resistance poses a significant challenge in treating and eliminating infections caused by NTM, particularly those belonging to the *Mycobacterium abscessus *complex* (*MABC) group. These infections are notorious for their resilience to standard antibiotics, making effective treatment more difficult. The *erm41* gene plays a pivotal role in macrolide resistance within the MABC group. Specifically, it is responsible for conferring resistance to macrolides in two of the three subspecies: *M. abscessus* and *M. bolletti*. However, *M. massiliense* subspecies tends to be macrolide-sensitive, leading to better treatment outcomes [13]. For individuals with *Mycobacterium abscessus* pulmonary disease, it is recommended to initiate a treatment plan based on susceptibility testing for macrolides and amikacin, rather than relying on empirical therapy [8]. In our case, the isolate was *Mycobacterium abscessus *subspp* abscessus, *which was resistant to macrolides and susceptible to aminoglycosides. This case report emphasizes that accurate identification of NTM, down to the species level, and specifically for *Mycobacterium abscessus*, to the subspecies level, plays a pivotal role in furnishing critical clinical and epidemiological insights. This precise identification aids in informed treatment decisions for patients.

For individuals diagnosed with *Mycobacterium abscessus* pulmonary disease, the Infectious Diseases Society of America (IDSA) recommends an initial treatment phase involving a multidrug regimen comprising a minimum of three active drugs, guided by in vitro susceptibility [8]. In individuals diagnosed with *Mycobacterium abscessus* pulmonary disease caused by strains lacking inducible or mutational resistance, IDSA recommends a multidrug treatment regimen that includes macrolides. For patients with *M. abscessus* pulmonary disease caused by strains exhibiting inducible or mutational macrolide resistance, IDSA suggests a macrolide-containing regimen, even if the macrolide is not considered an active drug in the multidrug regimen, due to its potential immunomodulatory properties. It’s important to note that *M. abscessus* infections can be life-threatening, and the use of macrolides may offer significant benefits. Amikacin, cefoxitin, imipenem, clarithromycin, linezolid, doxycycline, tigecycline, ciprofloxacin, and moxifloxacin are among the drugs that can be used against *Mycobacterium abscessus* [8].

Amikacin, a crucial drug in the treatment of *Mycobacterium abscessus* pulmonary disease, encounters resistance due to a specific mutation (A1408G) in the 16S rRNA (rrs) gene. This mutation has been linked to a high minimum inhibitory concentration (MIC) exceeding 64 μg/mL, particularly in patients with prior exposure to amikacin [14].

The absence of extensive research, coupled with differing drug availability, resource constraints, and variations in practice settings, has posed a challenge in reaching a unified agreement regarding the ideal duration of therapy. Existing literature indicates that most patients with *M. abscessus *have been treated for over 12 months. Treatment typically involves an initial phase that often includes parenteral drugs, followed by a longer phase utilizing oral and occasionally inhaled antibiotics [8].

## Conclusions

*Mycobacterium abscessus* complex is one of the known causes of iatrogenically acquired infections, most commonly following certain surgical procedures. In patients with long-term indwelling catheters in sterile sites, *Mycobacterium abscessus *complex is known to cause infections. Our case is a typical presentation of an iatrogenically acquired NTM infection by *Mycobacterium abscessus* subsp. abscessus. It is essential to identify the NTM as the treatment regimen differs according to the identification. Many laboratories do not have the facility to identify *Mycobacterium abscessus *up to the subspecies level, which is crucial for deciding the treatment. This emphasizes the importance of molecular methods like LPA, which will help to attain the identification as well as the susceptibility in a short time compared to conventional methods. In this case, since it was an iatrogenically acquired infection, this also emphasizes the importance of following hand hygiene, standard precautions, and hospital infection control policies.
